# T-bet is a key modulator of IL-23-driven pathogenic CD4^+^ T cell responses in the intestine

**DOI:** 10.1038/ncomms11627

**Published:** 2016-05-19

**Authors:** Thomas Krausgruber, Chris Schiering, Krista Adelmann, Oliver J. Harrison, Agnieszka Chomka, Claire Pearson, Philip P. Ahern, Matthew Shale, Mohamed Oukka, Fiona Powrie

**Affiliations:** 1 Translational Gastroenterology Unit, Experimental Medicine Division, Nuffield Department of Clinical Medicine, University of Oxford, John Radcliffe Hospital, Oxford OX3 9DU, UK; 2 The Kennedy Institute of Rheumatology, University of Oxford, Roosevelt Drive, Headington, Oxford OX3 7FY, UK; 3 Sir William Dunn School of Pathology, University of Oxford, Oxford OX1 3RE, UK; 4 Center for Immunity and Immunotherapies, Seattle Children's Research Institute, Seattle, Washington 98101, USA

## Abstract

IL-23 is a key driver of pathogenic Th17 cell responses. It has been suggested that the transcription factor T-bet is required to facilitate IL-23-driven pathogenic effector functions; however, the precise role of T-bet in intestinal T cell responses remains elusive. Here, we show that T-bet expression by T cells is not required for the induction of colitis or the differentiation of pathogenic Th17 cells but modifies qualitative features of the IL-23-driven colitogenic response by negatively regulating IL-23R expression. Consequently, absence of T-bet leads to unrestrained Th17 cell differentiation and activation characterized by high amounts of IL-17A and IL-22. The combined increase in IL-17A/IL-22 results in enhanced epithelial cell activation and inhibition of either IL-17A or IL-22 leads to disease amelioration. Our study identifies T-bet as a key modulator of IL-23-driven colitogenic responses in the intestine and has important implications for understanding of heterogeneity among inflammatory bowel disease patients.

## Abstract (web summary)

How transcription factor T-bet and Th17 cells contribute to colitis remains incompletely understood. Here the authors identify T-bet as a negative regulator of IL-23R pathway activation and show that T-bet deficient T cells drive colitogenic Th17 responses dependent on the cytokines IL-17A and IL-22.

## Graphical abstract

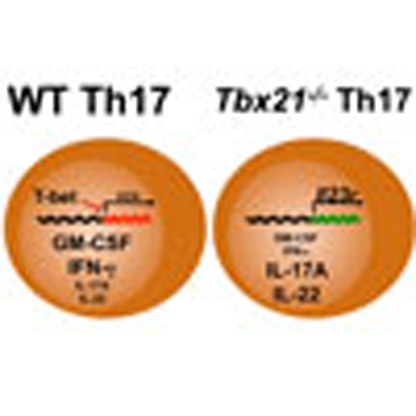

Th17 cells are enriched at mucosal sites, produce high amounts of IL-17A, IL-17F and IL-22, and have an essential role in mediating host protective immunity against a variety of extracellular pathogens^1^. However, on the dark side, Th17 cells have also been implicated in a variety of autoimmune and chronic inflammatory conditions, including inflammatory bowel disease (IBD)^2^. Despite intense interest, the cellular and molecular cues that drive Th17 cells into a pathogenic state in distinct tissue settings remain poorly defined.

The Th17 cell programme is driven by the transcription factor retinoid-related orphan receptor gamma-t (RORγt) (ref. 3), which is also required for the induction and maintenance of the receptor for IL-23 (refs 4, 5). The pro-inflammatory cytokine IL-23, composed of IL-23p19 and IL-12p40 (ref. 6), has been shown to be a key driver of pathology in various murine models of autoimmune and chronic inflammatory disease such as experimental autoimmune encephalomyelitis (EAE)^7^, collagen induced arthritis^8^ and intestinal inflammation^9^,^10^,^11^,^12^. Several lines of evidence, predominantly derived from EAE, suggest that IL-23 promotes the transition of Th17 cells to pathogenic effector cells^9^,^10^,^11^,^12^. Elegant fate mapping experiments of IL-17A-producing cells during EAE have shown that the majority of IL-17A^+^IFN-γ^+^ and IL-17A^−^IFN-γ^+^ effector cells arise from Th17 cell progeny^13^. This transition of Th17 cells into IFN-γ-producing ‘ex' Th17 cells required IL-23 and correlated with increased expression of T-bet. The T-box transcription factor T-bet drives the Th1 cell differentiation programme^14^ and directly transactivates the *Ifng* gene by binding to its promoter as well as multiple enhancer elements^15^. Indeed, epigenetic analyses have revealed that the loci for T-bet and IFN-γ are associated with permissive histone modifications in Th17 cells suggesting that Th17 cells are poised to express T-bet which could subsequently drive IFN-γ production^16^,^17^.

A similar picture is emerging in the intestine where IL-23 drives T-cell-mediated intestinal pathology which is thought to be dependent on expression of T-bet^18^ and RORγt (ref. 19) by T cells. In support of this we have recently shown that IL-23 signalling in T cells drives the emergence of IFN-γ producing Th17 cells in the intestine during chronic inflammation^20^. Collectively these studies suggest a model whereby RORγt drives differentiation of Th17 cells expressing high amounts of IL-23R, and subsequently, induction of T-bet downstream of IL-23 signalling generates IL-17A^+^IFN-γ^+^ T cells that are highly pathogenic. Indeed, acquisition of IFN-γ production by Th17 cells has been linked to their pathogenicity in several models of chronic disease^13^,^21^,^22^,^23^,^24^ and a population of T cells capable of producing both IL-17A and IFN-γ has also been described in intestinal biopsies of IBD patients^25^,^26^.

However, in the context of intestinal inflammation, it remains poorly defined whether the requirement for RORγt and T-bet reflects a contribution of Th17 and Th1 cells to disease progression or whether Th17 cells require T-bet co-expression to exert their pathogenic effector functions. Here, we use two distinct models of chronic intestinal inflammation and make the unexpected finding that T-bet is dispensable for IL-23-driven colitis. Rather the presence of T-bet serves to modify the colitogenic response restraining IL-17 and IL-22 driven pathology. These data identify T-bet as a key modulator of IL–23-driven colitogenic effector responses in the intestine and have important implications for understanding of heterogeneous immune pathogenic mechanisms in IBD patients.

## Results

### IL-23 promotes IL-17A^+^IFN-γ^+^ intestinal T cells

**Figure 1:**
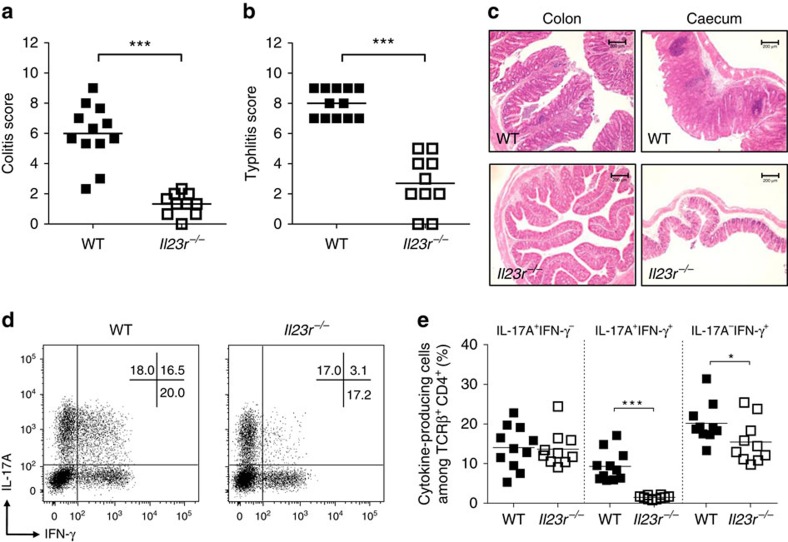
IL-23 signalling is required for bacteria-driven T-cell-dependent colitis and the emergence of IL-17A^+^IFN-γ^+^ T cells. C57BL/6 WT and *Il23r*^*−/−*^ mice were infected orally with *Hh* and received weekly i.p. injections of IL-10R blocking antibody. Mice were killed at 4 weeks post infection and assessed for intestinal inflammation. (**a**) Colitis scores. (**b**) Typhlitis sores. (**c**) Representative photomicrographs of colon and caecum (× 10 magnification; scale bars, 200 μM). (**d**) Representative flow cytometry plots of colonic lamina propria gated on viable CD4^+^ T cells. (**e**) Frequencies of IL-17A^+^ and/or IFN-γ^+^ CD4^+^ T cells present in the colon. Data represent pooled results from two independent experiments (*n*=12 for WT, *n*=10 for *Il23r*^*−/−*^). Bars are the mean and each symbol represents an individual mouse. *P<0.05, ***P<0.001 as calculated by Mann–Whitney *U* test.

Using the T cell transfer model of colitis, which involves the transfer of naïve CD4^+^ T cells into lymphopenic *Rag1*^*−/−*^ hosts^27^,^28^, we previously demonstrated that direct signalling of IL-23 into T cells promotes colitis and the emergence of IL-17A^+^IFN-γ^+^ T cells^20^. To assess the contribution of IL-23 signalling to the development of intestinal inflammation and differentiation of IL-17A^+^IFN-γ^+^ T cells in a lymphocyte replete setting, we induced colitis by oral infection with *Helicobacter hepaticus (Hh)* and concomitant administration of IL-10R blocking antibody (*Hh*+anti-IL-10R)^9^. This T-cell-dependent model of colitis is characterized by a mixed Th1/Th17 response and inflammation is blocked in *Il12p40*^*−/−*^ mice (deficient in both IL-12 and IL-23) but not *Il12p35*^*−/−*^ mice (deficient only in IL-12), inferring an essential role for IL-23 in disease pathogenesis. Consistent with this, we observed marked accumulation of IL-23R-expressing Th17 cells^29^ in the colon during the course of infection (Supplementary Fig. 1A,B). To assess the functional role of IL-23R signalling, we induced colitis in wild type (WT) or IL-23R-deficient (*Il23r*^*−/−*^) mice by infection with *Hh*+anti-IL-10R. As expected WT mice developed severe colitis and typhlocolitis characterized by cellular infiltration, broadening of crypts and epithelial hyperplasia (Fig. 1a–c). By contrast, *Il23r*^*−/−*^ mice showed only mild signs of inflammation in the colon and caecum. Genetic ablation of IL-23R did not affect the differentiation of IL-17A^+^IFN-γ^−^ T cells but led to a sixfold reduction in the frequency of IL-17A^+^IFN-γ^+^ T cells and a small decrease in IL-17A^−^IFN-γ^+^ T cells (Fig. 1d,e). Together, these data demonstrate that IL-23 is essential for the development of *Hh*-induced colitis and the emergence of IL-17A^+^IFN-γ^+^ T cells.

### IL-23 signals are dispensable for T-bet and RORγt expression

Evidence obtained from *in vitro* studies suggests that IL-23 drives the acquisition of IFN-γ production by Th17 cells in a T-bet-dependent manner^21^,^30^,^31^. In addition, IL-23 has been shown to enhance RORγt expression in naive CD4^+^ T cells transduced with IL-23R-expressing retrovirus^4^. Thus, IL-23 could drive the differentiation of IL-17A^+^IFN-γ^+^ T cells by virtue of its ability to induce and/or maintain expression of RORγt and T-bet. To address this, we generated mixed bone-marrow chimaeras by reconstituting irradiated *Rag1*^*−/−*^ mice with WT (CD45.1^+^) and *Il23r*^*−/−*^ (CD45.2^+^) bone marrow cells at 1:1 ratio. Following reconstitution of the haematopoietic system, we induced intestinal inflammation by infection with *Hh*+anti-IL-10R. This approach allowed us to investigate the phenotype of WT and *Il23r*^*−/−*^ CD4^+^ T cells exposed to the same inflammatory environment. Indeed, intestinal pathology in mixed bone-marrow chimaeras was indistinguishable from that in WT mice (Supplementary Fig. 2A and Fig. 1a). Both WT and *Il23r*^*−/−*^ CD4^+^ T cells were able to accumulate in the colon (Supplementary Fig. 2B) and we observed equivalent frequencies and total numbers of IL-17A^+^IFN-γ^−^ and IL-17A^−^IFN-γ^+^ cells among WT and *Il23r*^*−/−*^ T cells (Supplementary Fig. 2C,D). By contrast, differentiation of IL-17A^+^IFN-γ^+^ cells was significantly reduced in *Il23r*^*−/−*^ CD4^+^ T cells indicating that IL-23 signalling directly into T cells in a cell autonomous way is required for the emergence of this effector cell type^20^.

Next, we assessed the expression of RORγt and T-bet protein by cytokine producing T cells of WT and *Il23r*^*−/−*^ origin. As expected, IL-17A^+^IFN-γ^−^ cells expressed high amounts of RORγt and IL-17A^−^IFN-γ^+^ cells expressed high amounts of T-bet, whereas co-expression of both RORγt and T-bet was exclusive to IL-17A^+^IFN-γ^+^ T cells (Supplementary Fig. 2E–G). Noticeably, there were no discernible differences in RORγt or T-bet protein amounts between IL-17A or IFN-γ single producers of WT and *Il23r*^*−/−*^ origin (Supplementary Fig. 2G). In addition, the remaining IL-17A^+^IFN-γ^+^ cells detectable in T cells derived from *Il23r*^*−/−*^ bone marrow expressed equivalent amounts of T-bet and RORγt compared with their WT counterparts (Supplementary Fig. 2G). These results demonstrate that cell-intrinsic IL-23 signals are required for the emergence of IL-17A^+^IFN-γ^+^ T cells but are dispensable for the maintenance of RORγt and T-bet expression in intestinal CD4^+^ T cells under inflammatory conditions.

### RORγt but not T-bet is required for T cell transfer colitis

**Figure 2:**
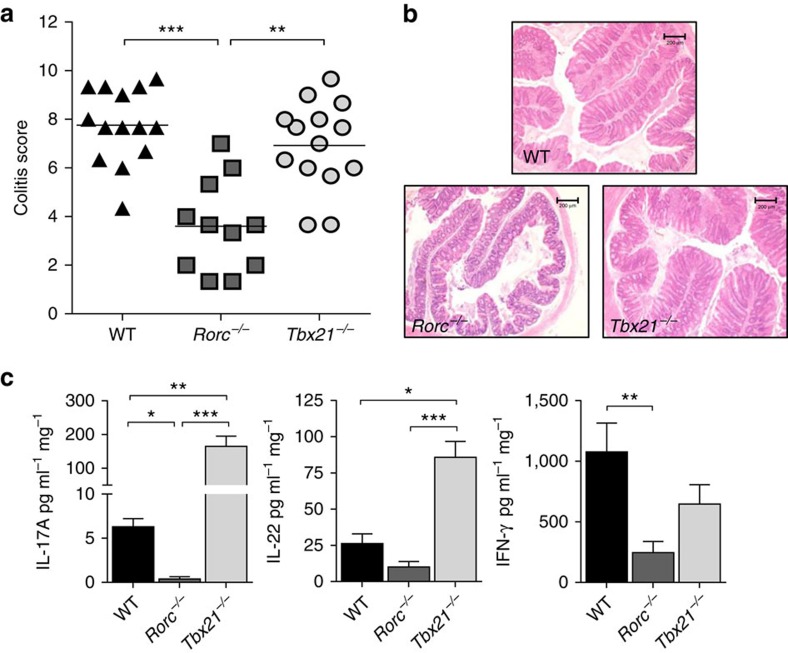
RORγt but not T-bet expression by CD4^+^ T cells is required for the development of T cell transfer colitis. C57BL/6 *Rag1*^*−/−*^ mice were injected i.p. with 4 × 10^5^ CD4^+^CD25^−^CD45RB^hi^ T cells from C57BL/6 WT, *Rorc*^*−/−*^ or *Tbx21*^*−/−*^ donors. Mice were killed when recipients of *Tbx21*^*−/−*^ T cells developed clinical signs of disease (4–6 weeks) and assessed for intestinal inflammation. (**a**) Colitis scores. (**b**) Representative photomicrographs of proximal colon sections (× 10 magnification; scale bars, 200 μM). (**c**) Concentration of cytokines released from colon explants into the medium after overnight culture. Data represent pooled results from two independent experiments (*n*=14 for WT, *n*=11 for *Rorc*^*−/−*^, *n*=14 for *Tbx21*^*−/−*^). Bars are the mean and each symbol represents an individual mouse. Bars are the mean and error bars represent s.e.m. *P<0.05, **P<0.01, ***P<0.001 as calculated by Kruskal–Wallis one-way ANOVA with Dunn's post-test.

To elucidate the impact of RORγt and T-bet expression on the colitogenic potential of T cells, we transferred *Rag1*^*−/−*^ mice with CD4^+^ T cells from C57BL/6 WT, *Tbx21*^*−/−*^ or *Rorc*^*−/−*^ donors and assessed the development of colitis. T cell transfer colitis is a useful model to dissect the contribution of adaptive immune cells to the development of the disease as genes can be selectively ablated in the T cell donors hence avoiding confounding effects on other immune populations. In keeping with previous reports^19^ *Rorc*^*−/−*^ T cells, which are deficient in both RORγ and RORγt, induced only mild colitis upon transfer into *Rag1*^*−/−*^ recipients (Fig. 2a,b). Consistent with the attenuated histological features, we detected reduced amounts of pro-inflammatory cytokines IL-17A, IL-22 and IFN-γ in colon explant cultures of *Rorc*^*−/−*^ T cell recipients (Fig. 2c). Strikingly, transfer of *Tbx21*^*−/−*^ CD4^+^ T cells led to the development of a similar degree of intestinal inflammation as transfer of WT T cells (Fig. 2a,b). Despite equivalent pathology, analysis of cytokine secretion in colon explant cultures revealed a distinct inflammatory signature in recipients of *Tbx21*^*−/−*^ T cells, characterized by significantly elevated amounts of the Th17 signature cytokines IL-17A and IL-22 (Fig. 2c). Surprisingly, transfer of *Tbx21*^*−/−*^ T cells only marginally affected IFN-γ amounts, suggesting that T-bet-independent IFN-γ secretion by T cells, or IFN-γ expressing innate cells, contribute to IFN-γ production in this setting.

Further analysis showed that dysregulated Th2 or Foxp3^+^ regulatory T cell (Treg cells) responses are unlikely to account for the difference in colitogenic potential of T-bet and RORγt-deficient T cells. We did not detect any significant differences in the frequency of Foxp3^+^ Treg cells, IL-4 or IL-13 expressing cells between *Rorc*^*−/−*^ and *Tbx21*^*−/−*^ T cells (Supplementary Fig. 3A–C).

### T-bet is dispensable for IL-17A^+^IFN-γ^+^ intestinal T cells

**Figure 3:**
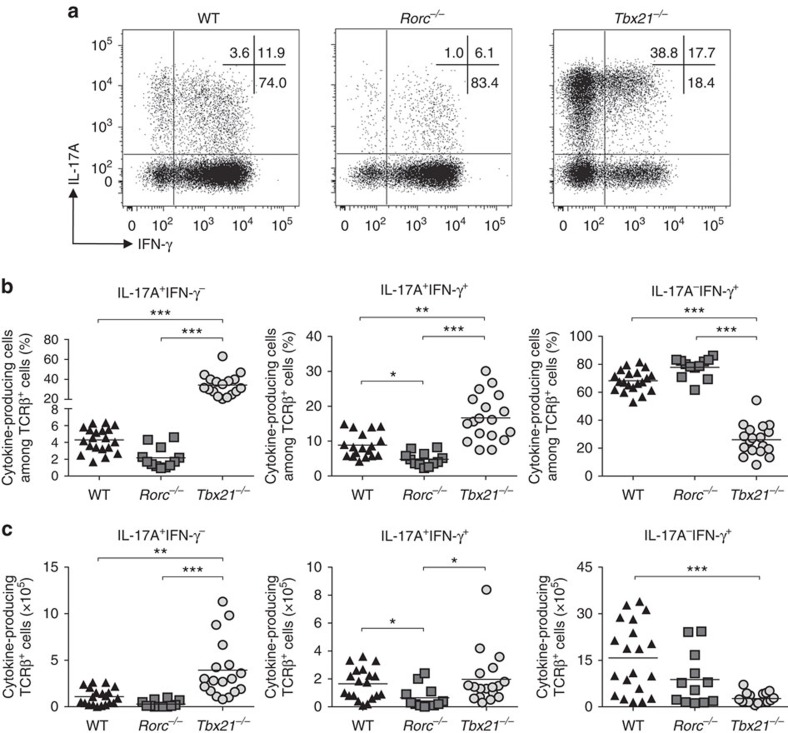
T-bet expression by CD4^+^ T cells is not required for the emergence of IL-17A^+^IFN-γ^+^ T cells. C57BL/6 *Rag1*^*−/−*^ mice were injected i.p. with 4x10^5^ CD4^+^CD25^−^CD45RB^hi^ T cells from C57BL/6 WT, *Rorc*^*−/−*^ or *Tbx21*^*−/−*^ donors. Mice were killed when recipients of *Tbx21*^*−/−*^T cells developed clinical signs of disease (4–6 weeks). (**a**) Representative plots of IL-17A and IFN-γ expression in colonic CD4^+^ T cells. (**b**) Frequencies of IL-17A^+^ and/or IFN-γ^+^ cells among colonic CD4^+^ T cells. (**c**) Total numbers of IL-17A^+^ and/or IFN-γ^+^ CD4^+^ T cells present in the colon. Data represent pooled results from three independent experiments (n=20 for WT, n=18 for *Tbx21*^*−/−*^, n=12 for *Rorc*^*−/−*^). Bars are the mean and each symbol represents an individual mouse. *P<0.05, **P<0.01, ***P<0.001 as calculated by Kruskal–Wallis one-way ANOVA with Dunn's post-test.

Next we addressed the impact of T-bet or RORγt-deficiency on intestinal CD4^+^ T cell differentiation. Consistent with their role in T helper cell development, deficiency in T-bet led to impaired differentiation of IFN-γ^+^ cells while the emergence of IL-17A^+^ cells was markedly reduced in RORγt-deficient T cells (Fig. 3a). The attenuated intestinal pathology observed in recipients of *Rorc*^*−/−*^ T cells (Fig. 2a) correlated with a significantly decreased frequency and total number of IL-17A^+^IFN-γ^+^ T cells in the colon (Fig. 3b,c). By contrast, T-bet deficiency did not affect the differentiation and accumulation of IL-17A^+^IFN-γ^+^ T cells (Fig. 3a–c), indicating that T-bet is dispensable for the generation of this effector cell population. Together, these data reinforce the requirement for RORγt in the generation of intestinal Th17 cells and provide evidence that T-bet is dispensable for the differentiation of intestinal IL-17A^+^IFN-γ^+^ T cells.

### T-bet deficiency promotes an exacerbated Th17-type response

**Figure 4:**
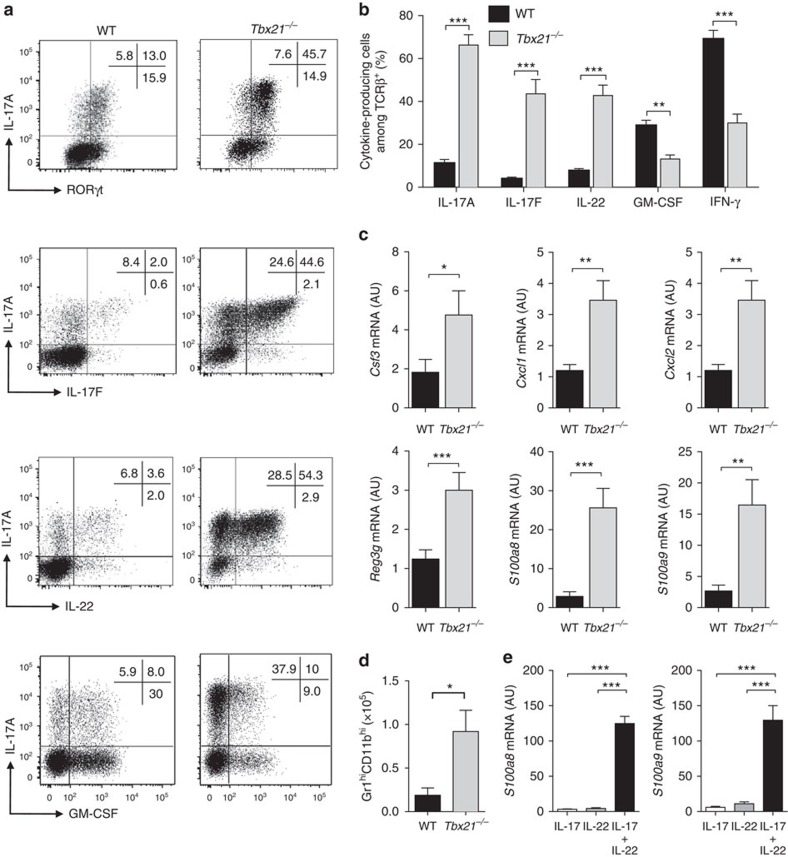
T-bet-deficient CD4^+^ T cells promote an exacerbated Th17-type inflammatory response. C57BL/6 *Rag1*^*−/−*^ mice were injected i.p. with 4x10^5^ CD4^+^CD25^−^CD45RB^hi^ T cells from C57BL/6 WT or *Tbx21*^*−/−*^ donors. Mice were killed when recipients of *Tbx21*^*−/−*^T cells developed clinical signs of disease (4–6 weeks). (**a**) Representative plots of cytokines and transcription factors in WT or *Tbx21*^*−/−*^ colonic CD4^+^ T cells. (**b**) Frequency of IL-17A^+^, IL-17F^+^, IL-22^+^, GM-CSF^+^ or IFN-γ^+^ colonic T cells in WT or *Tbx21*^*−/−*^. (**c**) quantitative reverse transcription PCR (qRT-PCR) analysis of mRNA levels of indicated genes in colon tissue homogenates. (**d**) Total number of neutrophils (CD11b^+^ Gr1^high^) in the colon. (**e**) Primary epithelial cells were isolated from the colon of steady state C57BL/6 *Rag1*^*−/−*^ mice and stimulated with 10 ng ml^−1^ cytokines for 4 h after which cells were harvested and analysed by qRT-PCR for the indicated genes. Data in **b**–**d** represent pooled results from two independent experiments (*n*=14 for WT, *n*=11 for *Tbx21*^*−/−*^). Bars are the mean and error bars represent s.e.m. Data in **e** are pooled results from four independent experiments, bars are the mean and error bars represent s.e.m. *P<0.05, **P<0.01, ***P<0.001 as calculated by Mann–Whitney *U* test.

Our transfer of *Tbx21*^*−/−*^ T cells revealed a striking increase in the frequency of IL-17A^+^IFN-γ^−^ cells (Fig. 3) and we reasoned that T-bet-deficiency could impact on Th17 cell cytokine production. Therefore, we transferred WT or *Tbx21*^*−/−*^ CD4^+^ T cells into *Rag1*^*−/−*^ recipients and measured the expression of RORγt, IL-17A, IL-17F and IL-22 by CD4^+^ T cells isolated from the colon. In agreement with our earlier findings, *Tbx21*^*−/−*^ T cells gave rise to significantly increased frequencies of RORγt-expressing T cells capable of producing IL-17A (Fig. 4a). Furthermore, T-bet deficiency also led to a dramatic expansion of IL-17F and IL-22-expressing cells, which constituted only a minor fraction in WT T cells (Fig. 4a,b). By contrast, the frequency of granulocyte-macrophage colony-stimulating factor (GM-CSF) and IFN-γ producing cells was significantly reduced in T-bet-deficient T cells as compared with WT T cells. When analysed in more detail we noted that the production of IL-17A, IL-17F and IL-22 increased specifically in T-bet-deficient IL-17A^+^IFN-γ^+^ T cells as compared with WT T cells whereas IFN-γ production decreased overall in the absence of T-bet as expected (Supplementary Fig. 4A). Similarly, GM-CSF production was also generally reduced in *Tbx21*^*−/−*^ CD4^+^ T cells further suggesting a shift in the qualitative nature of the T cell response.

Next, we compared the expression of genes that are downstream of IL-17A, IL-17F and IL-22 signalling in colon homogenates of *Rag1*^*−/−*^ recipients of WT or *Tbx21*^*−/−*^ T cells. Transcript expression of *Csf3*, *Cxcl1*, *Cxcl2*, *Reg3g*, *S100a8* and *S100a9* were significantly increased in the colon of *Rag1*^*−/−*^ recipients of *Tbx21*^*−/−*^ T cells (Fig. 4c). Consistent with this, we also found increased numbers of neutrophils in the colonic lamina propria of these mice (Fig. 4d). To test possible interactions between IL-22 and IL-17A on expression of these genes we isolated epithelial cells from the colon of *Rag1*^*−/−*^ mice and stimulated them with IL-17A, IL-22 or combination of both cytokines. We found a marked synergistic increase in the expression of the neutrophil chemoattractants *S100a8* and *S100a9* (ref. 32) messenger RNA (mRNA) following stimulation with IL-17A and IL-22 (Fig. 4e and Supplementary Fig. 4B). Together, our data demonstrate that colitis induced by *Tbx21*^*−/−*^ T cells is characterized by an exacerbated Th17-type response and that synergistic activity of IL-22 and IL-17A on epithelial cells may result in enhanced neutrophil recruitment and development of intestinal inflammation with distinct hall marks.

### T-bet-deficient CD4^+^ T cells are hyper-responsive to IL-23

**Figure 5:**
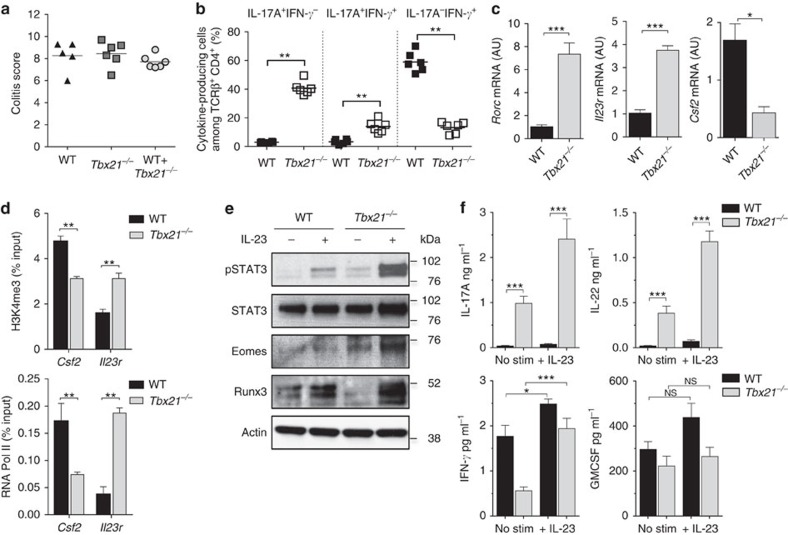
T-bet-deficient T cells are hyper-responsive to IL-23. C57BL/6 *Rag1*^*−/−*^ mice were injected i.p. with either WT or *Tbx21*^*−/−*^ CD4^+^CD25^-^CD45RB^hi^ T cells or a 1:1 mixtures of WT (CD45.1^+^) and *Tbx21*^*−/−*^(CD45.2^+^) CD4^+^CD25^−^CD45RB^hi^ T cells. Mice were killed when recipients of *Tbx21*^*−/−*^ T cells developed clinical signs of disease (∼5 weeks) and assessed for intestinal inflammation. (**a**) Colitis scores. (**b**) Frequencies of IL-17A^+^ and/or IFN-γ^+^ cells in the colon of mice receiving mix of WT and *Tbx21*^*−/−*^ CD4^+^ T cells. (**c**) CD4^+^ T cells were purified by flow cytometry from the inflamed colon of mice receiving a mix of WT and *Tbx21*^*−/−*^ CD4^+^ T cells and mRNA levels of indicated genes were analysed by quantitative reverse transcription PCR (qRT-PCR). (**d**) ChIP-qRT-PCR for the occupancy of H3K4me3 or RNA Pol II around the TSS of indicated genes in CD4^+^ T cells purified as described in **c**. (**e**) Immunoblot for pY705-STAT3, total STAT3, EOMES, Runx3 and Actin on whole cells lysates of CD4^+^ T cells purified as in **c**, left unstimulated or stimulated with IL-23 for 12 h. (**f**) Concentrations of indicated cytokines secreted into culture medium by CD4^+^ T cells purified and stimulated as described in **e**. Data in **a**–**c** and **f** represent results from one of two independent experiments (*n*=5 for WT, *n*=6 for *Tbx21*^*−/−*^, *n*=6 for WT+*Tbx21*^*−/−*^). Bars are the mean and each symbol represents an individual mouse. Bars are the mean and error bars represent s.e.m. Data in **d** are pooled results from two independent experiments, bars are the mean and error bars represent s.d. *P<0.05, **P<0.01, ***P<0.001 as calculated by Mann–Whitney *U* test.

Next, we sought to compare gene-expression signatures and functional properties of pathogenic T cells generated from WT and *Tbx21*^*−/−*^ T cells. To investigate this, we purified CD4^+^ T cells from the inflamed colon of *Rag1*^*−/−*^ mice that had been transferred with an equal number of WT (CD45.1^+^) and *Tbx21*^*−/−*^ (CD45.2^+^) T cells. Intestinal pathology of mice receiving WT and *Tbx21*^*−/−*^ T cells was indistinguishable from that in mice transferred with WT or *Tbx21*^*−/−*^ only T cells (Fig. 5a). Importantly, the increased differentiation of *Tbx21*^*−/−*^ T cell progeny into IL-17A^+^IFN-γ^+^ T cells was preserved in the co-transfer (Fig. 5b and Supplementary Fig. 5A) further supporting the notion that T-bet is dispensable for acquisition of IFN-γ production by Th17 cells. Comparison of gene-expression signatures showed that T-bet-deficient T cells had significantly higher expression of *Rorc, Il23r*, *Ccr6* and *Il1r1* indicating a specific cell-intrinsic role for T-bet in the repression of Th17 signature genes (Fig. 5c and Supplementary Fig. 5B). In addition, *Tbx21*^*−/−*^ T cells had upregulated expression of neutrophil chemoattractant *Cxcl3* and decreased expression of the gene encoding GM-CSF (*Csf2*). Interestingly, we found similar amounts of mRNA for *Il10* and *Tgfb3* (Supplementary Fig. 5B) suggesting that these cytokines do not contribute to the observed differences in pathogenic effector function between WT and *Tbx21*^*−/−*^ T cells.

We were intrigued by the fact that *Il23r* and *Csf2* appeared to be differentially expressed in WT versus *Tbx21*^*−/−*^ T cells and tested whether T-bet would be directly involved in regulating their transcription. We performed ChIP experiments in CD4^+^ T cells cultured *in vitro* under Th0 or Th1 conditions and analysed the recruitment of T-bet to the transcription start site (TSS) of *Csf2* and to a specific region in intron 3 of *Il23r* where T-bet binding has been detected according to two independent T-bet ChIP-Seq experiments^33^,^34^. To correlate T-bet binding with gene activity we analysed in parallel the recruitment of RNA polymerase II (RNA Pol II) as well as the occurrence of the open-chromatin-associated histone mark H3K4me3 at the TSS of *Csf2* and *Il23r*. T-bet was robustly recruited to *Csf2* and *Il23r* in Th1 cells and despite elevated amounts of H3Kme3 at both genes only *Csf2* showed enhanced transcriptional activity, as measured by the recruitment of RNA Pol II (Supplementary Fig. 5C). To further investigate the role of T-bet in the transcriptional regulation of *Csf2* and *Il23r* we performed ChIP experiments on progeny of WT and *Tbx21*^*−/−*^ CD4^+^ T cells sort-purified from T cell co-transfer experiments described above. In the absence of T-bet, the open-chromatin mark H3K4me3 was increased at the *Il23r* TSS as compared with WT cells and transcription of the *Il23r* genes was upregulated substantially in *Tbx21*^*−/−*^ progeny (Fig. 5d). This was in contrast to the *Csf2* gene where T-bet deficiency resulted in decreased occupancy of both H3K4me3 and RNA Poll II (Fig. 5d). These data provide a molecular basis for our functional studies and reveal opposing functions of T-bet leading to positive regulation of *Csf2* but negative regulation of *Il23r* expression.

Given the increased amounts of *Il23r* expression, we reasoned that the exacerbated Th17 response induced by transfer of *Tbx21*^*−/−*^ T cells could be the result of enhanced responsiveness to IL-23. Stimulation of colonic WT T cells with IL-23 resulted in phosphorylation of STAT3 and this was greatly enhanced in colonic *Tbx21*^*−/−*^ T cells (Fig. 5e), suggesting that T-bet-deficient T cells are hyper-responsive to IL-23 due to higher expression of IL-23R. Analysis of cytokine production by sort-purified colonic T cells showed increased secretion of IL-17A and IL-22 in unstimulated and IL-23-activated *Tbx21*^*−/−*^ compared with WT T cells (Fig. 5f). IL-23-stimulated IFN-γ production was also increased in *Tbx21*^*−/−*^ T cells relative to unstimulated controls whereas IL-23 had no effect on GM-CSF production in WT or T-bet deficient T cells (Fig. 5f).

To investigate the potential mechanism responsible for T-bet-independent IFN-γ production, we measured induction of Eomesodermin (Eomes) and Runt-related transcription factor 3 (Runx3) downstream of IL-23 stimulation in sort-purified WT and *Tbx21*^*−/−*^ colonic effector T cells. Eomes is a paralogue to T-bet with a known role in regulating IFN-γ production in both CD4^+^ and CD8^+^ T cells^35^,^36^,^37^. Eomes was detected in unstimulated *Tbx21*^*−/−*^ T cells and its expression was further induced in response to IL-23 stimulation. In contrast, Eomes protein amounts were at the limit of detection in unstimulated and IL-23-stimulated WT T cells (Fig. 5e). In line with these results, we found that intracellular expression of Eomes was highly upregulated in colonic T-bet-deficient effector T cells as compared with their WT counterparts (Supplementary Fig. 6A). However, <10% of IL-17A^+^IFN-γ^+^ *Tbx21*^*−/−*^ T cells expressed Eomes compared with ∼75% of IL-17A^−^IFN-γ^+^ T cells (Supplementary Fig. 6B) suggesting that this factor might drive IFN-γ expression specifically in the latter population. Runx3 has been shown to promote IFN-γ secretion directly either through binding to the *Ifng* promoter^38^ or indirectly through induction of Eomes^39^. Runx3 was induced in WT and T-bet-deficient T cells with greater fold induction observed in *Tbx21*^*−/−*^ T cells (Fig. 5e). Thus, Eomes and Runx3 appear to be induced downstream of IL-23R signalling in *Tbx21*^*−/−*^ CD4^+^ T cells and could potentially contribute to T-bet-independent IFN-γ production.

### T-bet-deficient colitis depends on IL-23, IL-17A and IL-22

**Figure 6:**
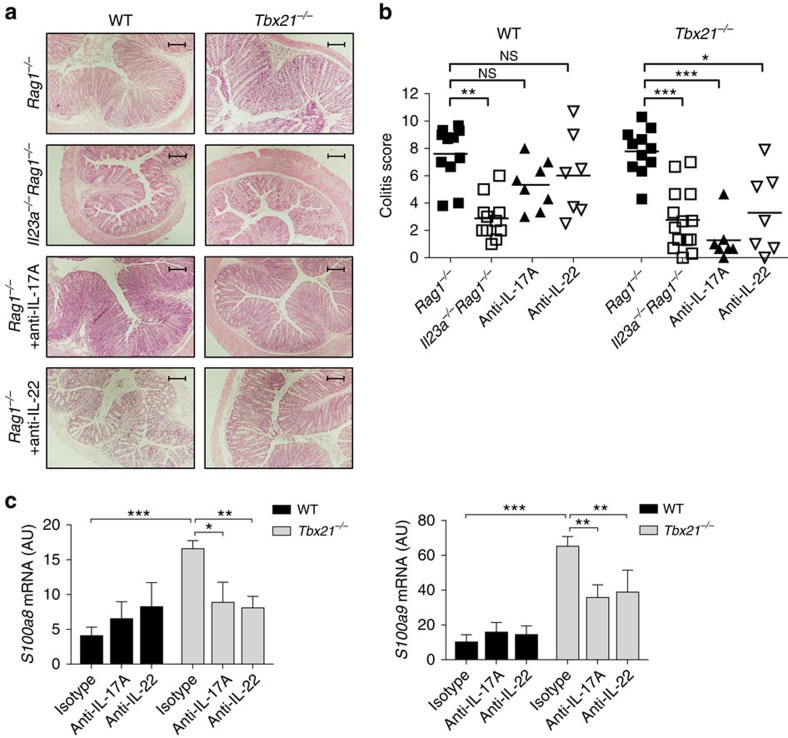
Colitis induced by T-bet-deficient T cells is dependent on IL-23, IL-17A and IL-22. C57BL/6 *Rag1*^*−/−*^ mice or *Il23a*^*−/−*^*Rag1*^*−/−*^ were injected i.p. with 4 × 10^5^ CD4^+^CD25^-^CD45RB^hi^ T cells from C57BL/6 WT or *Tbx21*^*−/−*^ donors. *Rag1*^*−/−*^ recipients received weekly injections of blocking anti-IL-17A, anti-IL-22 or isotype control mAbs. Mice were killed when recipients of isotype control antibody developed clinical signs of disease (4–6 weeks). (**a**) Representative photomicrographs of proximal colon sections from the indicated experimental groups (× 10 magnification; scale bars, 200 μM). (**b**) Colitis score. (**c**) Primary epithelial cells were isolated from the colon of steady state C57BL/6 *Rag1*^*−/−*^ mice and stimulated with supernatant of IL-23-stimulated WT or *Tbx21*^*−/−*^ CD4^+^ T cells sort-purified from *Rag1*^*−/−*^ recipients. Supernatants were added at 20% of total cell culture volume in combination with indicated blocking antibodies. Cells were harvested after 4 h and mRNA levels of indicated genes were analysed by quantitative reverse transcription PCR (qRT-PCR). Data in **b** represents pooled results from two independent experiments. Bars are the mean and each point represents an individual mouse. Data in **c** are pooled results from four independent experiments, bars are the mean and error bars represent s.e.m. *P<0.05, **P<0.01, ***P<0.001 as calculated by Kruskal–Wallis one-way ANOVA with Dunn's post-test.

To investigate whether IL-23 has a functional role in the induction of colitis by T-bet-deficient T cells, *Rag1*^*−/−*^ and *Il23a*^*−/−*^*Rag*^*−/−*^ mice were transferred with WT or *Tbx21*^*−/−*^ T cells. As expected, transfer of WT or *Tbx21*^*−/−*^ T cells induced severe intestinal inflammation in *Rag1*^*−/−*^ hosts. By contrast, colitis was significantly decreased when WT or *Tbx21*^*−/−*^ T cells were transferred into IL-23 deficient *Il23a*^*−/−*^*Rag1*^*−/−*^ recipients (Fig. 6a,b). To test which effector cytokines were required for colitis induced by T-bet-deficient T cells, we transferred cohorts of *Rag1*^*−/−*^ recipients with WT or *Tbx21*^*−/−*^ T cells and administered neutralizing antibodies to IL-17A or IL-22. Although neutralization of IL-17A or IL-22 did not significantly reduce inflammation in recipients of WT T cells, blockade of either cytokine abrogated intestinal pathology in *Rag1*^*−/−*^ mice transferred with *Tbx21*^*−/−*^ CD4^+^ T cells (Fig. 6a,b). To confirm that IL-17A/IL-22 produced by CD4^+^ T cells are indeed pathogenic factors in *Tbx21*^*−/−*^ driven T cell transfer colitis, we stimulated intestinal epithelial cells with supernatant of IL-23-activated WT and *Tbx21*^*−/−*^ T cells. Compared with WT supernatant, the supernatant of *Tbx21*^*−/−*^ T cells induced a potent epithelial cell response (Fig. 6c). Similar to the *in vivo* blockade experiments, neutralization of IL-17A or IL-22 was only effective in *Tbx21*^*−/−*^ T cells suggesting that augmented cytokine production may be a major driver in colitis induced by T-bet-deficient T cells. Taken together these results show that IL-23 promotes a distinct Th17-dependent pathogenic T cell response in the absence of T-bet.

## Discussion

In the present study we show that bacteria-driven colitis is associated with the IL-23-dependent emergence of IFN-γ-producing Th17 cells co-expressing RORγt and T-bet. Strikingly, while RORγt is required for the differentiation of IFN-γ-producing Th17 cells and induction of colitis, T-bet is dispensable for the emergence of IL-17A^+^IFN-γ^+^ T cells and intestinal pathology. Our results show that instead of a mandatory role in the colitogenic response, the presence of T-bet modulates the qualitative nature of the IL-23-driven intestinal inflammatory response. In the presence of T-bet, IL-23-driven colitis is multifunctional in nature and not functionally dependent on either IL-17A or IL-22. By contrast, in the absence of T-bet a highly polarized colitogenic Th17 cell response ensues which is functionally dependent on both IL-17A and IL-22. T-bet-deficient T cells are hyper-responsive to IL-23 resulting in enhanced STAT3 activation and downstream cytokine secretion providing a mechanistic basis for the functional changes. These data newly identify T-bet as a key modulator of IL-23-driven colitogenic CD4^+^ T cell responses.

Contrary to our expectations T-bet expression by CD4 T cells was not required for their pathogenicity. In keeping with the negative effect of T-bet on Th17 differentiation^40^,^41^,^42^, we observed highly polarized Th17 responses in T-bet-deficient intestinal T cells. Early studies demonstrated that IFN-γ could suppress the differentiation of Th17 cells^40^ and thus the reduced IFN-γ production by *Tbx21*^*−/−*^T cells could facilitate Th17 cell generation. However, our co-transfer studies revealed unrestrained Th17 differentiation of *Tbx21*^*−/−*^ T cells even in the presence of WT T cells, suggesting a cell autonomous role for T-bet-mediated suppression of the Th17 programme. Indeed, the role of T-bet as a transcriptional repressor of the Th17 cell fate has been described recently. For example, T-bet physically interacts with and sequesters Runx1, thereby preventing Runx1-mediated induction of RORγt and Th17 cell differentiation^43^. In addition, T-bet binds directly to and negatively regulates expression of many Th17-related genes^15^,^34^ and we identified *IL23r* to be repressed in a T-bet-dependent manner. In line with this we show here that T-bet-deficient intestinal T cells express higher amounts of *Il23r* as well as *Rorc*. This resulted in enhanced IL-23-mediated STAT3 activation and increased production of IL-17A and IL-22. It has also been suggested that T-bet activation downstream of IL-23R signalling is required for pathogenic IL-23-driven T cell responses^43^,^44^. However, we did not find a role for IL-23 in the induction and/or maintenance of T-bet expression and colitis induced by T-bet-deficient T cells was IL-23 dependent. Collectively, these findings demonstrate that T-bet deficiency leads to unrestrained expansion of colitogenic Th17 cells, which is likely mediated through enhanced activation of the IL-23R-STAT3 pathway.

The observation that T-bet-deficient T cells retain their colitogenic potential is in stark contrast to earlier studies. Neurath *et al*.^18^ convincingly showed that adoptive transfer of *Tbx21*^*−/−*^ CD4^+^ T cells into severe combined immunodeficiency (SCID) recipients failed to induce colitis and this correlated with reduced IFN-γ and increased IL-4 production. Another study revealed that IL-4 plays a functional role in inhibiting the colitogenic potential of *Tbx21*^*−/−*^ T cells, as recipients of *Stat6*^*−/−*^*Tbx21*^*−/−*^ T cells developed severe colitis^37^. Importantly, the intestinal inflammation that developed in recipients of *Stat6*^*−/−*^*Tbx21*^*−/−*^ T cells could be blocked by administration of IL-17A neutralizing antibody, suggesting that the potent inhibitory effect of IL-4/STAT6 signals on Th17 differentiation normally prevent colitis induced by *Tbx21*^*−/−*^ T cells^37^. Various explanations could account for the discrepancy between our study and those earlier findings. First, in contrast to the published reports, we used naïve *Tbx21*^*−/−*^ CD4^+^ T cells from C57BL/6 mice instead of BALB/c mice. An important difference between *Tbx21*^*−/−*^ CD4^+^ T cells from these genetic backgrounds appears to be their differential susceptibility to suppression by IL-4/STAT6 signals. We found that transfer of *Tbx21*^*−/−*^ T cells induced IL-17A-dependent colitis despite increased frequencies of IL-4-expressing cells in the intestine. This discrepancy may be due to higher amounts of IL-4 produced by activated CD4^+^ T cells from BALB/c versus C57BL/6 mice^45^, leading to the well-described Th2-bias of the BALB/c strain^45^. Second, differences in the composition of the intestinal microbiota between animal facilities can have a substantial effect on skewing CD4^+^ T cells responses. In particular, the *Clostridium*-related segmented filamentous bacteria (SFB) have been shown to drive the emergence of IL-17 and IL-22 producing CD4^+^ T cells in the intestine^46^. Importantly, the ability of naïve CD4^+^ T cells to induce colitis is dependent on the presence of intestinal bacteria, as germ-free mice do not develop pathology upon T cell transfer^47^. In line with this, we previously described that colonization of germ-free mice with intestinal microbiota containing SFB was necessary to restore the development of colitis^47^. Since our *Rag1*^*−/−*^ colony is SFB^+^ and the presence of SFB was not reported in the previous studies, it is possible that differences in SFB colonization status contributed to the observed differences in pathogenicity of *Tbx21*^*−/−*^ T cells.

It is important to note that T-bet-deficient T cells did not induce more severe colitis than WT T cells but rather promoted a distinct mucosal inflammatory response. Colitis induced by WT T cells is characterized by a multifunctional response with high amounts of IFN-γ and GM-CSF and a lower IL-17A and IL-22 response. Consistent with this, we have shown that blockade of GM-CSF abrogates T cell transfer colitis^48^ as well as bacteria-driven intestinal inflammation^49^ in T-bet sufficiency whereas blockade of IL-17A or IL-22 fails to do so. By contrast T-bet deficiency leads to production of high amounts of IL-17A and IL-22 in the colon and neutralization of either was sufficient to reduce intestinal pathology. Our *in vitro* experiments suggest that IL-17A and IL-22 synergise to promote intestinal epithelial cell responses, which may in part explain the efficacy of blocking IL-17A or IL-22 in colitis induced by T-bet-deficient T cells. A similar synergistic interplay has been described in the lung where IL-22 served a tissue protective function in homeostasis but induced airway inflammation in the presence of IL-17A (ref. 50). This highlights the complexity of the system in health and disease, and the need for a controlled production of both cytokines. We describe here only one mechanism of how IL-17A/IL-22 induce a context-specific epithelial cell response that potentially impacts on the order or composition of immune cell infiltration. Overall, these results provide a new perspective on T-bet, revealing its role in shaping the qualitative nature of the IL-23-driven colitogenic T cell response.

We also describe here the unexpected finding that a substantial proportion of T-bet-deficient intestinal T cells retain the ability to express IFN-γ. To investigate the potential mechanisms responsible for T-bet-independent IFN-γ production by intestinal CD4^+^ T cells we focused on two transcription factors, Runx3 and Eomes. Runx3 has been shown to promote IFN-γ expression directly through binding to the *Ifng* promoter^38^ and Eomes is known to compensate for IFN-γ production in T-bet-deficient Th1 cells^37^. We found IL-23-mediated induction of Runx3 protein in WT and *Tbx21*^*−/−*^ T cells isolated from the intestine, thus identifying Runx3 downstream of IL-23R signalling. By contrast, we could only detect Eomes protein and its induction by IL-23 in T-bet-deficient but not WT T cells. Thus, Runx3 and Eomes are activated in response to IL-23 in T-bet-deficient cells and are likely to be drivers of T-bet-independent IFN-γ production. In support of this we found that the majority of T-bet-deficient IL-17A^−^IFN-γ^+^ T cells expressed Eomes. However, only a minor population of IL-17A^+^IFN-γ^+^ T cells stained positive for Eomes, suggesting the existence of alternative pathways for IFN-γ production by Th17 cells. Intriguingly, a recent study identified Runx3 and Runx1 as the transcriptional regulators critical for the differentiation of IFN-γ-producing Th17 cells^51^. The author's demonstrated that ectopic expression of Runx transcription factors was sufficient to induce IFN-γ production by Th17 cells even in the absence of T-bet. These findings, combined with our data on Runx3 activation downstream of IL-23R signalling strongly suggest that Runx3 rather than Eomes is driving IFN-γ expression by intestinal Th17 cells.

We have not formally addressed the role of IFN-γ in colitis driven by T-bet-deficient T cells. A recent report by Zimmermann *et al*.^52^ found that antibody-mediated blockade of IFN-γ ameliorates colitis induced by WT or T-bet-deficient T cells suggesting IFN-γ also contributes to the colitogneic response mediated by T-bet-deficient T cells as originally described for WT T cells^53^,^54^. By contrast with our results the Zimmerman study found that IL-17A blockade exacerbated colitis following transfer of *Tbx21*^*−/−*^ T cells. The reason for the differential role of IL-17A in the two studies is not clear but it is notable that the Zimmerman study was performed in the presence of co-infection with SFB and *Hh*, and this strong inflammatory drive may alter the pathophysiological role of particular cytokines. Together the data indicate that T-bet deficiency in T cells does not impede their colitogenic activity but that the downstream effector cytokines of the response are context dependent.

In conclusion, our data further underline the essential role for IL-23 in intestinal inflammation and demonstrate that T-bet is an important modulator of the IL–23-driven effector T cell response. The colitogenic T cell response in a T-bet sufficient environment is multifunctional with a dominant GM-CSF and IFN-γ response. By contrast T-bet-deficient colitogenic responses are dominated by IL-17A and IL-22-mediated immune pathology. These results may have significant bearing on human IBD where it is now recognized that differential responsiveness to treatment may reflect considerable disease heterogeneity. As such, identification of suitable biomarkers such as immunological parameters, that allow stratification of patient groups, is becoming increasingly important^55^. Genome-wide association studies have identified polymorphisms in loci related to innate and adaptive immune arms that confer increased susceptibility to IBD. Among these are Th1 (*STAT4*, *IFNG* and *STAT1*) as well as Th17-related genes (*RORC*, *IL23R* and *STAT3*) (refs 56, 57). Thus, detailed profiling of the T cell response in IBD patients may help identify appropriate patient groups that are most likely to benefit from therapeutic blockade of certain effector cytokines. Finally, our studies highlight the importance of IL-23 in the intestinal inflammatory hierarchy and suggest that IL-23 could be an effective therapeutic target across a variety of patient groups.

## Methods

### Mice

Wild type C57BL/6, C57BL/6 *Tbx21*^*−/−*^ (obtained from Jackson Laboratories) congenic B6.SJL-Cd45, C57BL/6 *Rag1*^*−/−*^, C57BL/6 *Rorc*^*−/−*^ (obtained from Dan Littman) C57BL/6 *Il23r*^*−/−*^, C57BL/6.IL-23R^gfp/+^ (obtained from Mohamed Oukka) and C57BL/6 *Il23a*^*−/−*^*Rag1*^*−/−*^ (obtained from Dan Cua) mice were bred and maintained under specific pathogen-free conditions in accredited animal facilities at the University of Oxford. All procedures were conducted in accordance with the UK Scientific Procedures Act of 1986. All experimental protocols were approved by the UK Home Office and the Ethical Review Committee of the Oxford University Medical Sciences Division. Both female and male mice were used in experiments and animals were assigned randomly to experimental groups. Each cage contained animals of all the different experimental groups whenever possible.

Mice were negative for Helicobacter spp. and other known intestinal pathogens and were more than 6 weeks old when first used for T cell transfer or when used as recipients/donors in mixed bone-marrow chimera experiments.

### T cell transfer colitis

For naïve T cell transfer colitis, 4 × 10^5^ CD4^+^CD25^−^CD45RB^hi^ T cells were injected i.p. into *Rag1*^*−/−*^ or *Il23a*^*−/−*^*Rag1*^*−/−*^ recipients. In co-transfer experiments, 2 × 10^5^ CD4^+^CD25^−^CD45RB^hi^ T cells from each source were mixed and injected i.p. into *Rag1*^*−/−*^ hosts. Where indicated, mice were injected with anti-IL-17A (1 mg per mouse every 3 days, clone 17F3 BioXCell), anti-IL-22 (0.125 mg per mouse every 3 days, Genentech, clone 8E11) or isotype control for the duration of the experiment. Mice were killed at indicated time points or killed when weight loss approached 20% of the original body weight at the start of the experiment.

### Induction of *Hh*/anti-IL-10R colitis

Mice were fed 1 × 10^8^ cfu *Hh* by oral gavage with a 22G curved needle on days 0 and 1 of the experiment. In some experiments, mice also received 1 mg of an anti-IL-10R blocking antibody by i.p. injection once weekly starting at the day of *Hh* infection.

### Generation of mixed bone-marrow chimeras

Bone marrow isolated from *Il23r*^*−/−*^ mice was mixed at a 1:1 ratio with bone marrow taken from B6.SJL-Cd45 mice and injected intravenously into gamma-irradiated (5.5 Gy, 550Rad) C57BL/6 *Rag1*^*−/−*^recipients and chimaeras were used in experiments >8 weeks after injection.

### Histological assessment of intestinal inflammation

Proximal, mid- and distal-colon samples were fixed in buffered 10% formalin solution. Paraffin-embedded sections were cut (5 mm) and stained with haematoxylin and eosin, and inflammation was scored in a blinded fashion using a previously described scoring system^58^. Briefly, each sample was graded semiquantitatively from 0 to 3 for the four following criteria: degree of epithelial hyperplasia and goblet cell depletion; leucocyte infiltration in the lamina propria; area of tissue affected; and the presence of markers of severe inflammation such as crypt abscesses, submucosal inflammation and ulcers. Scores for each criterion were added to give an overall inflammation score for each sample of 0–12. The total colonic score was calculated as the average of the individual scores from the sections of proximal-, mid- and distal-colon.

### Isolation of leucocyte subpopulations and flow cytometry

Cell suspensions from spleen, mesenteric lymph nodes (MLN) and the lamina propria were prepared as described previously^59^ and first incubated with anti-CD16/CD32 (eBioscience) to prevent nonspecific binding. Single cell suspensions were stained with antibodies against CD4, CD11b, CD25, GR1, TCR-β, CD45.1, CD45.2, IL-4, IL-13, IL-17A, IL-22, IFN-γ, EOMES, RORγt, Foxp3, (all from eBioscience), CD45RB, IL-17F and T-bet (all from Biolegend). Intracellular staining was performed as follows: cells were restimulated for 4 h with 0.1 μg ml^−1^ phorbol 12-myristate 13-acetate (PMA) and 1 μg ml^−1^ ionomycin (both Sigma Aldrich) plus Brefeldin A (eBioscience), washed and incubated with anti-CD16/CD32. Cells were washed and stained for surface markers indicated above and fixed in eBioscience Fix/Perm buffer, followed by permeabilization in eBioscience permeabilization buffer for 1 h in the presence of antibodies. Cells were acquired with a BD LSR2 and analysis was performed with FlowJo (Tree Star) software

### Colon explant cultures

Small pieces of colon (∼5 mm of mid-colon) were isolated, rinsed in PBS/BSA and weighed. Colon explants were cultured overnight in Roswell Park Memorial Institute medium (RPMI; Invitrogen) containing 10% FCS, 2 mM L-glutamine, 0.05 mM 2-mercaptoethanol and 100 U ml^−1^ each of penicillin and streptomycin (complete media) in 24-well tissue culture plates. Cytokine amounts in the supernatants were determined by Bender assay according to the manufacturers' protocol (eBioscience) and concentrations normalized to the weight of the explants.

### T cell cultures

CD4^+^TCR-β^+^CD45.1^+^ (WT progeny) and CD4^+^TCR-β^+^CD45.1^−^ (*Tbx21*^*−/−*^ progeny) T cells were sort-purified from the colon of C57BL/6 *Rag1*^*−/−*^ recipient mice. Cells were either lysed for quantitative PCR (qPCR) or plated at 2 × 10^5^ cells per well in flat bottomed 96-well plates in RPMI (Invitrogen) containing 10% FCS, 2 mM l-glutamine, 0.05 mM 2-mercaptoethanol and 100 U ml^−1^ each of penicillin and streptomycin (complete media). IL-23 was added at 20 ng ml^−1^ (R&D) for 12 h after which cells were harvested and lysed for immunoblot analysed. Supernatants were collected and cytokine secretion quantified by Bender assay (eBioscience) as per the manufacturers' protocol.

### Epithelial cell cultures

Primary epithelial cells were isolated from steady state colon of C57BL/6 *Rag1*^*−/−*^ mice. Briefly, colons were cut open longitudinally, washed in cold PBS to remove colon content and cut into 1 cm pieces. Epithelial cells were liberated by a 10-min wash with RPMI containing 10% FCS and 5 mM EDTA in a shaking incubator. Supernatant was filtered, cells pelleted at 1,500 r.p.m. and plated at 2 × 10^5^ cells per well in flat bottom 96-well plates in complete media. Recombinant IL-17 and IL-22 (Biolegend) were added for 4 h at the concentration indicated after which cells were harvested and lysed for qPCR.

In some experiments, CD4^+^TCR-β^+^CD45.1^+^ (WT progeny) and CD4^+^TCR-β^+^CD45.1^−^ (*Tbx21*^*−/−*^ progeny) T cells were sort-purified from the colon of C57BL/6 *Rag1*^*−/−*^ recipient mice. Cells were stimulated with IL-23 (10 ng ml^−1^) for 12 h, the supernatant collected and added at 20% of total culture volume to epithelial cells in combination with blocking antibodies as indicated: anti-IL-17A (10 μg ml^−1^, clone 17F3 BioXCell), anti-IL-22 (10 μg ml^−1^, Genentech) or isotype control. Cells were stimulated for 4 h after which cells were harvested and lysed for qPCR.

### qPCR

RNA was extracted according to the manufacturers' protocol (RNeasy, Qiagen) and cDNA synthesis was performed using Superscript III reverse transcription and Oligo dT primers (both from Invitrogen). qPCR reactions were performed using TaqMan Gene Expression Assays and normalized to hypoxanthine-guanine phosphoribosyltransferase (HPRT; all from Applied Biosystems). Samples were assayed in duplicate on a Bio-Rad CFX96 RT-qPCR or ABI Vii machine and differences were calculated using the 2ΔC(t) method.

### Total protein extracts and immunoblot analysis

Total protein extracts were prepared by lysing cells in protein lysis buffer (20 mM Tris pH 8.0, 300 mM NaCl, 10% Glycerol, 1% NP-40) for 30 min on ice. After centrifugation at full speed to pellet debris, equal amounts of protein were resolved by SDS–polyacrylamide gel electrophoresis and analysed with anti-pY705-STAT3 (clone D3A7, Cat. Nr. 9145, 1/1,000 dilution, Cell Signaling), anti-STAT3 (clone 79D7, Cat. Nr. 4904, 1/1,000 dilution, Cell Signaling), anti-EOMES (clone 21Mags8, Cat. Nr. 14-4876-80, 1/500 dilution, eBiosciences), anti-Actin (clone AC-15, Cat. Nr. A5541, 1/1,0000 Sigma) or anti-Runx3 (1/1,000, gift from Yoram Groner). Images in Fig. 5e have been cropped for presentation. Full size images are presented in Supplementary Fig. 7A–E.

### Chromatin immunoprecipitation

For *in vitro* ChIP assays, naïve CD4^+^CD25^−^CD44^−^CD62L^+^ T cells were sort-purified from spleens and lymphnodes of steady state C57BL/6 mice. Cells were plated at 3x10^5^ cells per well in complete media in 48-well plates coated with anti-CD3 (5 μg ml^−1^). Antibodies and cytokines were added at the following concentrations: anti-CD28 (2 μg ml^−1^) and IL-2 (100 U ml^−1^, Peprotech) for Th0 or anti-CD28 (2 μg ml^−1^), IL-2 (100 U ml^−1^), IL-12 (10 ng ml^−1^, Peprotech) and anti-IL-4 (10 μg ml^−1^, Biolegend) for Th1. Cells were cultured for 36 h and stimulated with fresh IL-12 (10 ng ml^−1^) for the last 4 h. For *ex vivo* ChIP assays, CD4^+^TCR-β^+^CD45.1^+^ (WT progeny) and CD4^+^TCR-β^+^CD45.1^−^ (*Tbx21*^*−/−*^ progeny) T cells were sort-purified from the colon of C57BL/6 *Rag1*^*−/−*^ recipient mice.

ChIP assays were performed as described^60^. Briefly, cells were washed once with PBS and fixed with 1% paraformaldehyde (Sigma) for 10 min at room temperature. 125 mM of Tris pH 7 was added to stop the reaction. Cells were pelleted, washed, lysed in sonication buffer (10 mM Tris-HCl pH 8.0, 100 mM NaCl, 1 mM EDTA, 0.5 mM EGTA, 0.1% Na-Deoxycholate, 0.5% Na-Lauroylsarcosine; all from Sigma) and sonicated using a Bioruptor Pico (Diagenode) to obtain a fragment size of around 500 base pairs. Lysates were centrifuged at full speed for 5 min at 4 °C and an aliquot (10%) was taken at this point as input control DNA. Remaining lysates were incubated with antibodies pre-bound to Protein G magnetic beads (Live Technologies) at 4 °C on a rotator overnight. The following antibodies were used, T-bet (Cat. Nr. sc-21003, 3 ug per IP, Santa Cruz), RNA Pol II (sc-899, 2 ug per IP, Santa Cruz) or H3K4me3 (Pab-003-050, 3 ug per IP, Diagenode). Beads were then placed on a magnet, washed five times with wash buffer (50 mM Hepes-KOH pH 7.6, 500 mM LiCl, 1 mM EDTA, 1% NP-40, 0.7% Na-deoxycholate; all from Sigma) and once with Tris-EDTA (TE) containing 50 mM NaCl. After the final wash, beads were resuspended in elution buffer (1 × TE containing 2% SDS) and reverse cross-linking performed at 65 °C in a shaking heat block overnight. Reverse cross-linking was similarly performed on input control DNA. The immunoprecipitated DNA fragments and input control DNA were purified using QIAquick PCR Purification Kit (Qiagen) as per the manufacturers' protocol and then analysed by quantitative reverse transcription PCR on a ABI Vii machine with SYBR Premix Ex Taq II master mix (Takara Bio) and the following primers: locus encoding *Csf2* TSS, 5′-agagaaaggctaaggtcctgagga-3′ and 5′-ccaggaggttcagggcttcttt-3', locus encoding *Il23r* TSS, 5′-agggctctgctgacatttccat-3′ and 5′-acagctgcctctatgtatctcagga-3′, or locus encoding part of *Il23r* intron 3, 5′-gtcactgagcacctgtcccttc-3′ and 5′-aaacccctcaaccaacagcagg-3′.

### Statistical analysis

Where appropriate the Mann–Whitney *U* test or Student's *t*-test was used. For comparison of more than two groups a Kruskal–Wallis one-way ANOVA followed by a Dunn's *post hoc* test or one-way ANOVA followed by a Bonferroni multiple comparison test was performed. All statistical analysis was calculated in Prism (Graphpad). Differences were considered to be statistically significant when *P*≤0.05.

### Data availability

The data that support the findings of this study are available from the corresponding authors on request.

## Additional information

**How to cite this article:** Krausgruber, T. *et al*. T-bet is a Key Modulator of IL-23-driven Pathogenic CD4^+^ T cell Responses in the Intestine. *Nat. Commun.* 7:11627 doi: 10.1038/ncomms11627 (2016).

## Supplementary Material

Supplementary Information Supplementary Figures 1-7
