# Supplementary material for: T-bet is a key modulator of IL-23-driven pathogenic CD4+ T cell responses in the intestine
Source: Nat Commun. 2016 May 19;7:11627. doi: 10.1038/ncomms11627 (PMC4874038; doi:10.1038/ncomms11627)
Supplement: Supplementary Information — Supplementary Figures 1-7 [file ncomms11627-s1.pdf]

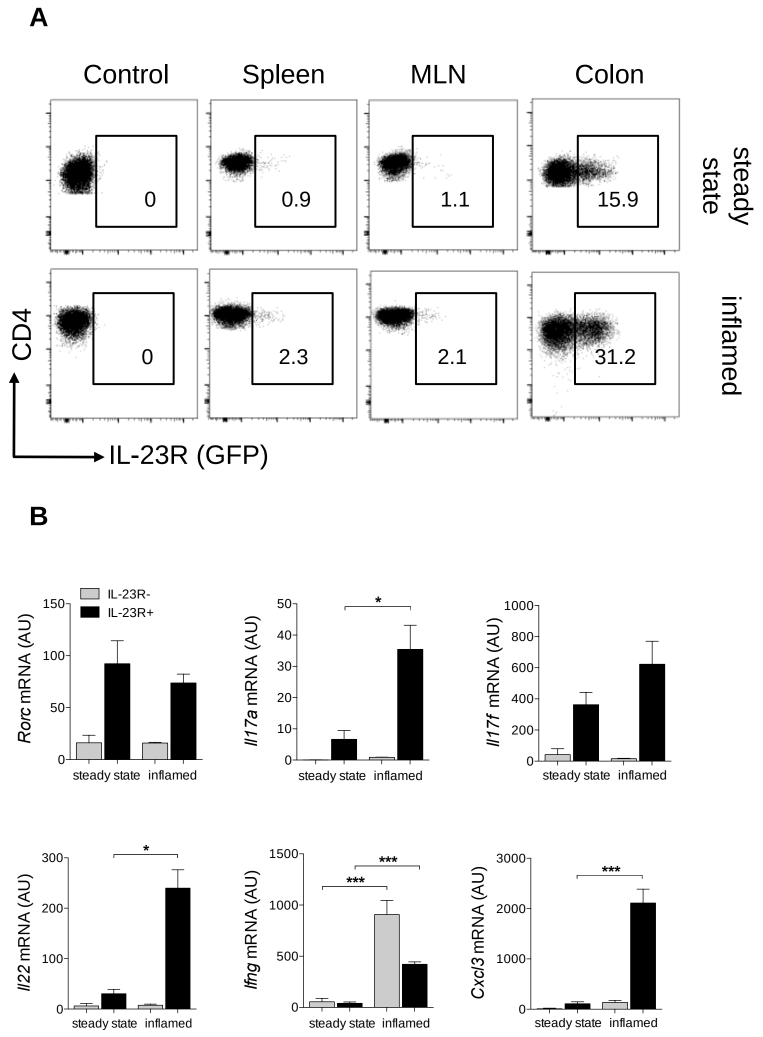

Supplementary Fig. 1

IL-23R-expressing CD4$^+$ T cells with a Th17 signature accumulate in bacteria-driven colitis

Heterozygous C57BL/6 IL-23R-GFP knock-in mice were infected orally with *Helicobacter hepaticus* and received weekly i.p. injections of IL-10R blocking antibody. Mice were sacrificed 2 weeks post infection **(A)** Representative plots of IL-23R(GFP) expression among CD4$^+$ T cells from the spleen, MLN and colon of steady state or inflamed mice. GFP-negative WT mice were used as staining control. Numbers in gates represent frequencies. **(B)** qRT-PCR analysis of mRNA levels of indicated genes in FACS-purified IL-23R(GFP)$^+$ and IL-23R(GFP)$^-$ CD4$^+$ T cells from the colon of steady state or inflamed mice (n = 4 per group).

Data represent pooled results from two independent experiments. Bars are the mean and error bars represent the standard error of the mean (s.e.m). **P*<0.05, ****P*<0.001 as calculated by Mann-Whitney U test.

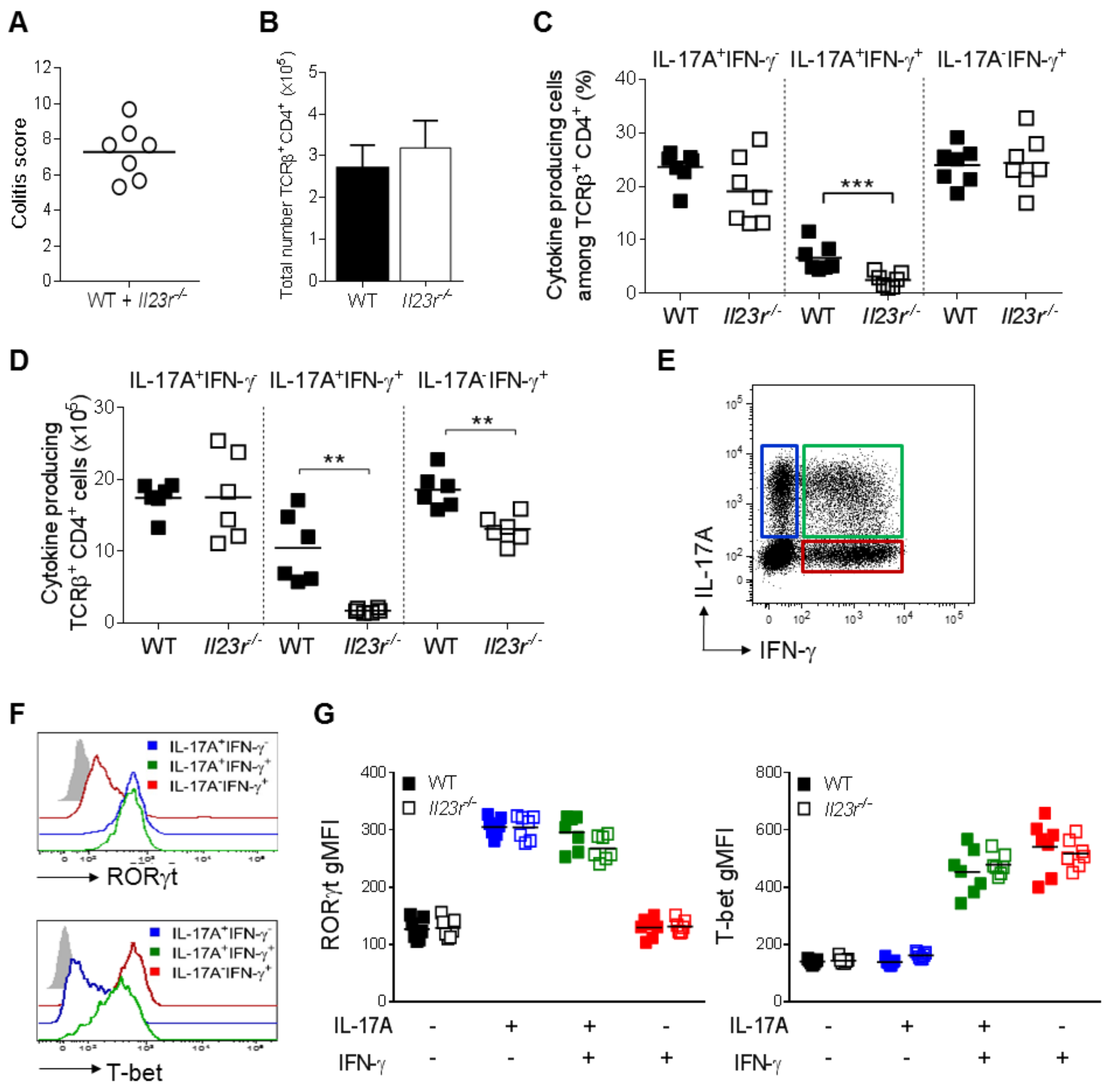

Supplementary Fig. 2

Expression of RORγt and T-bet in CD4^{+} T cells is not controlled by IL-23 signalling

Mixed bone marrow chimaeras were generated by irradiation of C57BL/6 *Rag1*$^{-/-}$ mice followed by i.v. injection of a 1:1 mixture of WT (CD45.1^{+}) and *Il23r*$^{-/-}$ (CD45.2^{+}) bone marrow cells. After 8 weeks, mice were infected orally with *Helicobacter hepaticus* and received weekly injections of IL-10R blocking antibody. Mice were sacrificed 4 weeks post infection and assessed for intestinal inflammation. **(A)** Colitis Score. **(B)** Total numbers of WT and *Il23r*$^{-/-}$ CD4^{+} T cells present in the colon. **(C)** Frequencies and **(D)** total numbers of IL-17A^{+} and/or IFN-γ^{+} cells among WT or *Il23r*$^{-/-}$ CD4^{+} T cells in the colon. **(E)** Representative FACS plots showing IL-17A^{+}IFN-γ^{-} (blue), IL-17A^{+}IFN-γ^{+} (green) and IL-17A^{-}IFN-γ^{+} (red) populations among WT CD4^{+} T cells in the colon. **(F)** Representative histogram of RORγt **(top)** and T-bet **(bottom)** expression among the cytokine-expressing cells gated as described in **E**. **(G)** Geometric mean fluorescence intensity (gMFI) of RORγt **(left)** and T-bet **(right)** among cytokine-expressing CD4^{+} T cells of WT or *Il23r*$^{-/-}$ origin gated as indicated.

Data represent results from a single experiment (n = 7). Bars are the mean and each symbol represents an individual mouse. Bars are the mean and error bars represent s.e.m. ****P*<0.001 as calculated by Mann-Whitney U test

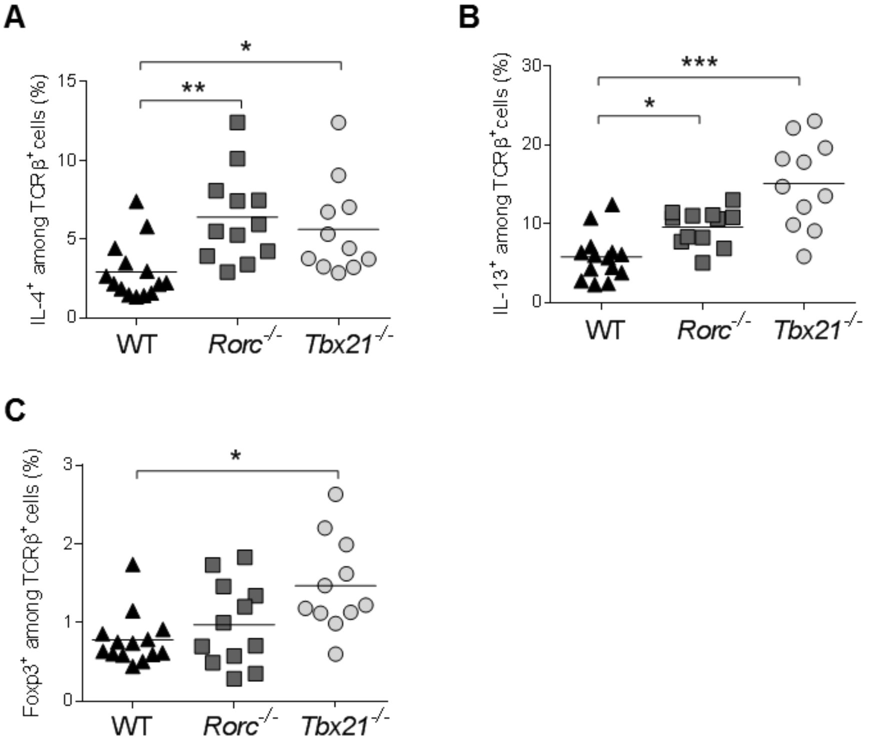

Supplementary Fig. 3

Effects of T-bet or RORγt deficiency on Th2 and Treg differentiation

Colitis was induced in C57BL/6 *Rag1*$^{-/-}$ mice as described in Figure 3. **(A)** Frequency of IL-4 producing cells, **(B)** IL-13 producing cells and **(C)** Foxp3^{+} cells among CD4^{+} T cells in the colon. Data represent pooled results from two independent experiments (n=14 for WT, n=11 for *Tbx21*$^{-/-}$, n=12 for *Rorc*$^{-/-}$). Bars are the mean and each point represents an individual mouse. **P*<0.05, ***P*<0.01, ****P*<0.001 as calculated by Kruskal-Wallis one-way ANOVA with Dunn's post-test.

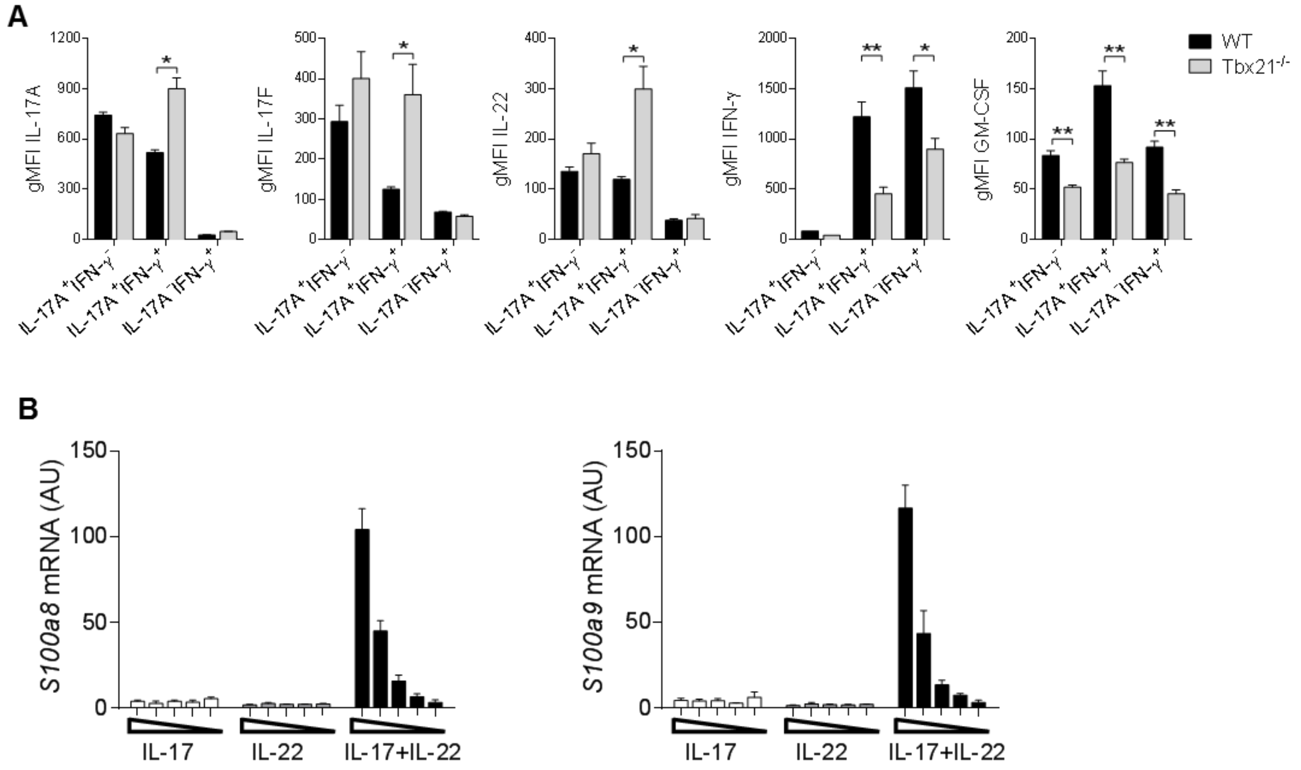

Supplementary Fig. 4

Gut epithelial cells are synergistically activated by IL-17A and IL-22

C57BL/6 *Rag1*$^{-/-}$ mice were injected i.p. with 4x10^{5} CD4^{+}CD25^{-}CD45RB^{hi} T cells from C57BL/6 WT or *Tbx21*$^{-/-}$ donors. Mice were sacrificed when recipients of *Tbx21*$^{-/-}$ T cells developed clinical signs of disease (4-6 weeks). **(A)** gMFI of IL-17A, IL-17F, IL-22, IFN-γ or GM-CSF among IL-17A^{+} and/or IFN-γ^{+} colonic T cells. **(B)** Primary epithelial cells were isolated form the colon of steady state C57BL/6 *Rag1*$^{-/-}$ mice. Cells were stimulated with IL-17A, IL-22 or combination of both (5 point dose response ranging from 10 ng/ml, 1 ng/ml, 100 pg/ml, 10 pg/ml to 1pg/ml) for 4 hrs after which cells were harvested and analysed by qRT-PCR for the indicated genes.

Data in A represent pooled results from two independent experiments (n=14 for WT, n=11 for *Tbx21*$^{-/-}$).

Bars are the mean and error bars represent s.e.m. Data in B represent results from one of three independent experiments, bars are the mean and error bars represent s.d. *P<0.05, **P<0.01, as calculated by Mann-Whitney U test

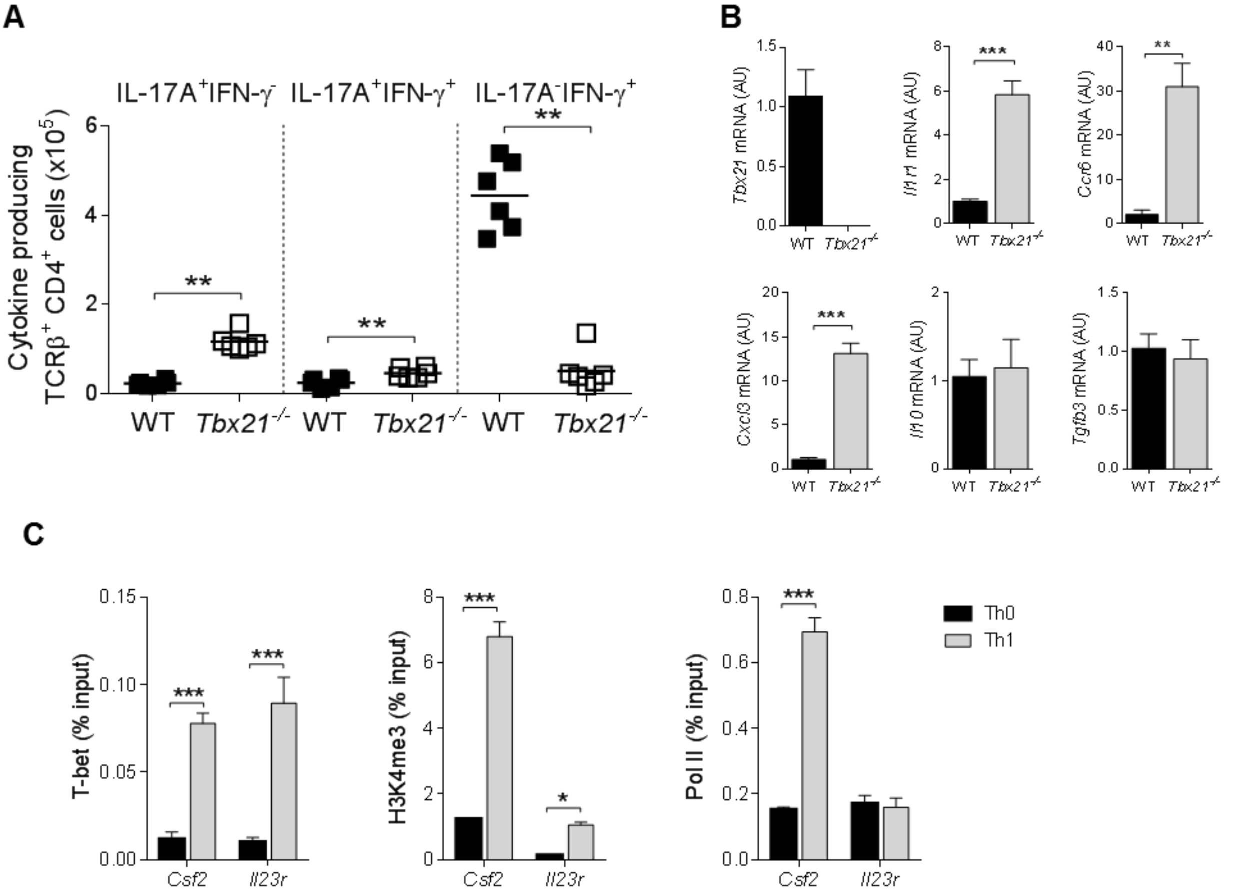

Supplementary Fig. 5

T-bet controls *Csf2* and *Il23r* expression in *in vitro* differentiated Th1 cells

C57BL/6 *Rag1*$^{-/-}$ mice were injected i.p. with 2x10^{5} CD4^{+}CD25^{-}CD45RB^{hi} T cells from C57BL/6 WT (CD45.1^{+}) and *Tbx21*$^{-/-}$ (CD45.2^{+}) donors. Mice were sacrificed when recipients of *Tbx21*$^{-/-}$ T cells developed clinical signs of disease (~5 weeks). **(A)** Total numbers of WT and *Tbx21*$^{-/-}$ CD4^{+} T cells present in the colon. **(B)** CD4^{+} T cells were purified by flow cytometry from the inflamed colon and mRNA levels of indicated genes analysed by qRT-PCR. **(C)** Naïve CD4^{+}CD25^{-}CD44^{-}CD62L^{+} T cells were cultured under Th0 or Th1 conditions for 36h and stimulated with fresh IL-12 (10 ng/ml) for the last 4h. ChIP-qRT-PCR for the occupancy of T-bet (TSS of *Csf2*, locus encoding part of intron 3 of *Il23r*), H3K4me3 or RNA Pol II around the TSS of indicated genes was analysed.

Data in A-B represent results from one of two independent experiments (n=6 for WT+*Tbx21*$^{-/-}$). Bars are the mean and each symbol represents an individual mouse. Bars are the mean and error bars represent s.e.m. Data in C are pooled results from three independent experiments, bars are the mean and error bars represent s.e.m. **P*<0.05, ***P*<0.01, ****P*<0.001 as calculated by Mann-Whitney U test.

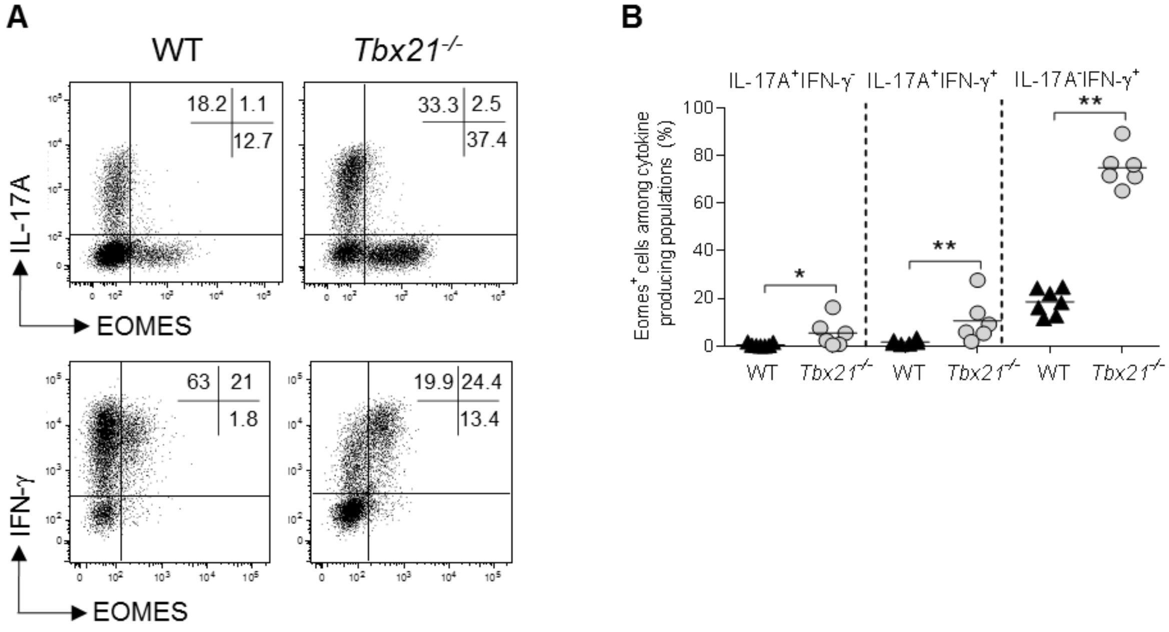

Supplementary Fig. 6

EOMES is upregulated in IFN-γ-producing T cells in the absence of T-bet

C57BL/6 *Rag1*$^{-/-}$ mice were injected i.p. with 4x10^{5} CD4^{+}CD25^{-}CD45RB^{hi} T cells from C57BL/6 WT or *Tbx21*$^{-/-}$ donors. Mice were sacrificed when recipients of *Tbx21*$^{-/-}$ T cells developed clinical signs of disease (4-6 weeks). **(A)** Representative plots of cytokine-producing EOMES^{+} colonic CD4^{+} T cells. **(B)** Frequencies of EOMES-expressing cells among IL-17A^{+} and/or IFN-γ^{+} CD4^{+} T cells in the colon.

Data represents results from one of two independent experiments (n=7 for WT, n=6 for *Tbx21*$^{-/-}$). Bars are the mean and each point represents an individual mouse. *P<0.05, **P<0.01, ***P<0.001 as calculated by Mann-Whitney U test.

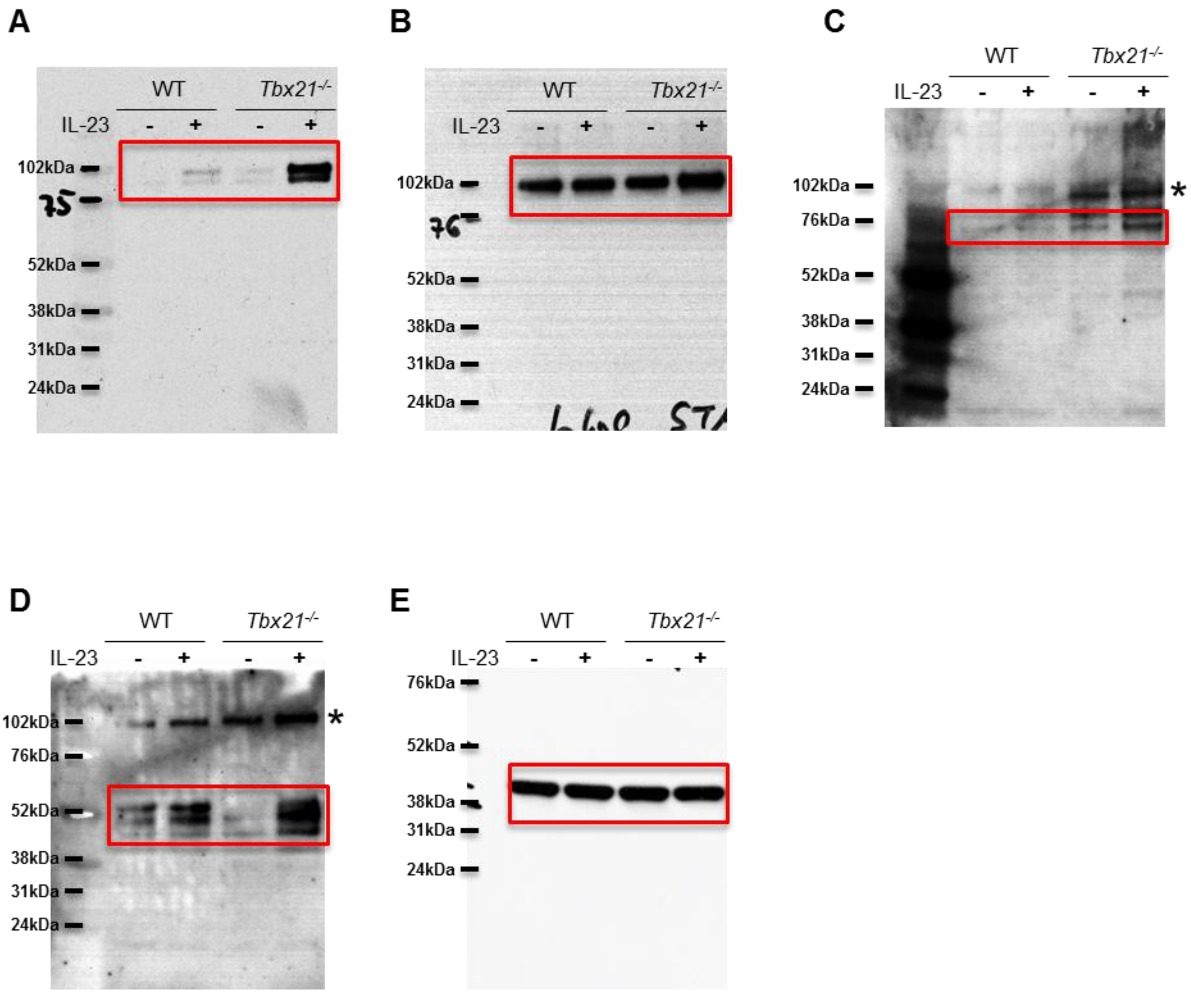

Supplementary Fig. 7

Full scans of immunoblots shown in Figure 5E

CD4$^+$ T cells were purified by flow cytometry from the inflamed colon of mice receiving mix of WT and *Tbx21*$^{-/-}$ CD4$^+$ T cells. After sorting, cells were left unstimulated or stimulated with IL-23 for 12 hrs. Immunoblot for **(A)** pY705-STAT3, **(B)** total STAT3, **(C)** EOMES, **(D)** Runx3 and **(E)** Actin on whole cell lysates. Cropped immunoblots presented in Figure 5E are indicated as read squares.
* depicts unspecific binding based on predicted molecular weight and information on splice variants.
